# Exogenous human OKSM factors maintain pluripotency gene expression of bovine and porcine iPS-like cells obtained with STEMCCA delivery system

**DOI:** 10.1186/s13104-018-3627-8

**Published:** 2018-07-27

**Authors:** Jesica R. Canizo, Camila Vazquez Echegaray, Doris Klisch, Juan F. Aller, Dante A. Paz, Ricardo H. Alberio, Ramiro Alberio, Alejandra S. Guberman

**Affiliations:** 1 Departamento de Producción Animal, INTA EEA Balcarce, Buenos Aires, Argentina; 2 Departamento de Química Biológica, Facultad de Ciencias Exactas y Naturales, Universidad de Buenos Aires, Buenos Aires, Argentina; 3 CONICET—Universidad de Buenos Aires, Instituto de Química Biológica (IQUIBICEN), Buenos Aires, Argentina; 4 School of Biosciences, University of Nottingham, Nottingham, LE12 5RD UK; 5 Departamento de Biodiversidad y Biología Experimental, Facultad de Ciencias Exactas y Naturales, Universidad de Buenos Aires, Buenos Aires, Argentina; 6 Instituto de Fisiología, Biología Molecular y Neurociencias (IFIBYNE), Universidad de Buenos Aires - CONICET, Buenos Aires, Argentina; 7 Facultad de Ciencias Agrarias, Universidad Nacional de Mar del Plata, Mar del Plata, Argentina; 8 Departamento de Fisiología y Biología Molecular y Celular, Facultad de Ciencias Exactas y Naturales, Universidad de Buenos Aires, Buenos Aires, Argentina

**Keywords:** iPS-like cells, Bovine and porcine fibroblasts, STEMCCA, Lentiviral vectors, Reprogramming

## Abstract

**Objectives:**

The use of induced pluripotent stem (iPS) cells as an alternative to embryonic stem cells to produce transgenic animals requires the development of a biotechnological platform for their generation. In this study, different strategies for the generation of bovine and porcine iPS cells were evaluated. Lentiviral vectors were used to deliver human factors OCT4, SOX2, KLF4 and c-MYC (OKSM) into bovine and porcine embryonic fibroblasts and different culture conditions were evaluated.

**Results:**

Protocols based on the integrative lentiviral vector STEMCCA produced porcine iPS-like cells more efficiently than in bovine cells. The iPS-like cells generated displayed stem cell features; however, expression of exogenous factors was maintained along at least 12 passages. Since inactivation of the exogenous factors is still a major bottleneck for establishing fully reprogrammed iPS cells, defining culture conditions that support endogenous OKSM expression is critical for the efficient generation of farm animals’ iPS cells.

**Electronic supplementary material:**

The online version of this article (10.1186/s13104-018-3627-8) contains supplementary material, which is available to authorized users.

## Main text

### Introduction

Since the first reports showing over-expression of OKSM transcription factors in mouse [1] and human [2, 3] cells to generate iPS cells (iPSCs), many attempts have been performed with bovine [4–7] and porcine cells [8, 9], albeit with limited success. In bovine, the use of retro and lentiviral vectors has been widely reported [10–14], one report describes the use of non-integrative vectors [15] and two studies report transposon-based delivery systems [7, 16]. A major limitation of current protocols for bovine and porcine iPSC generation is that in all cases the cells under the reprogramming process fail to silence the exogenous factors [8, 17]. Therefore, only partially reprogrammed b-iPS like cells and p-iPS like cells have been reported so far. Culture media used so far in farm animals iPSC generation do not sustain endogenous expression of pluripotent transcription factors [4], and therefore other culture conditions must be studied.

In human, cell culture conditions for embryonic stem cells (ESCs) and iPSCs have evolved from feeder-dependent and feeder-free medium to defined medium on defined extracellular matrix (ECM). In addition, chemical approaches have been developed to dramatically improve (> 200-fold) the efficiency of iPSC generation from human fibroblasts within 7 days of treatment [18], suggesting that some of these principles could be used with farm animal derived cells.

The main objective of this study was to establish a biotechnological platform for the generation of fully reprogrammed bovine and porcine iPSC using different strategies. Lentiviral vectors were used for introducing reprogramming factors human OKSM (h-OKSM) and different culture media were tested during the reprogramming of bovine and porcine embryonic fibroblasts (BEF and PEF, respectively).

### Methods

#### Cell culture, lentiviral particles stock preparation, transduction and reprogramming in FGF plus KSR medium

See Additional file 1.

#### Transduction and reprogramming of PEF in SB43 medium

iPS like cells were generated from PEF from 3rd passage (See Additional file 1, Fig. S1a). Fibroblasts were plated onto 6-well plates at a density of 100,000 cells/well for PEF and transduced with cryopreserved concentrated lentiviral particles (See Additional file 1). PEF were transduced twice (×2) at 24 h intervals. In each transduction run, 1.5 µL of STEMCCA stock and 7 µg/ml of Polybrene were added with 1.5 ml of complete DMEM, a medium that promotes fibroblast growth. PEF were passaged 72 h approximately after the last transduction, depending on the confluence, and seeded into Geltrex-coated 6-well plates with SB43 medium [18] at different densities (10,000 and 20,000 cells per well). Two densities are commonly tested to avoid over confluence. Medium was changed daily, and colonies were picked manually using a needle firstto detach them, and a p10 pipette after to pick them upThen,each picked colony wasdisaggregated completely or partially using TrypLE (INVITROGEN) in a well of a 4-well plate with 100 µl of the enzyme.TrypLE was inactivated adding an equal amount of FCS, cells were harvested by centrifugation and plated onto MEF feeder layer (see Additional file 1).

#### Transduction and reprogramming of BEF in SB43 medium

In a first attempt of reprogramming BEF with SB43 medium we proceeded as mentioned before for PEF. Since we couldn’t obtain iPSC-like colonies, we decided to add more runs of transduction on cryopreserved 2× transduced BEF. Cells were thaw and seeded at a density of 25,000 cells/well in a 6-well plate. The next day, cells were transduced 3 more times in a 24 h interval (See Additional file 1, Fig. S1b) and split at two densities (2500 and 5000 cells/well in a 6-well plate coated with Geltrex) at day 3. These cells were first cultured in complete DMEMto promote cell proliferation and switched to SB43 medium at the time they reached 20–30% of confluence as is recommended in [19]. SB43 medium was replaced every other day and cells were monitored daily. If cells reach high confluence while iPSC colonies emerge, cells should be passaged with EDTA at some point during the 2 weeks as is mentioned in [19]. Clonally expansion of b-iPS like cells was performed as mentioned above for p-iPS like cells.

#### Characterization of iPS like cells

See Additional file 1.

### Results

#### STEMCCA lentiviral vector transduction allowed bovine and porcine fibroblasts to initiate the reprogramming process

In a first run of experiments, transduced BEF and PEF were plated on MEF feeder layer and cultured in DMEM/F12 (20% KSR, 20 ng/ml FGF) at day 4 post-transduction. Morphology changes of porcine and bovine cells were detected at day 10 (See Additional file 2, Fig. S2a). Although bovine cells arrested growth, we obtained colonies from porcine cells. Porcine iPS like cells were obtained in all the conditions tested (with and without sodium butyrate and different MOI of STEMCCA), but as shown in Additional file 2, Fig. S2b, apoptotic cells and globular structures could be seen in most colonies at day 18, suggesting differentiation. Moreover, these cells could not be expanded by picking colonies manually, suggesting a loss of the self-renewal ability (See Additional file 2, Fig. S2c). In addition to the loss of the characteristic pluripotent stem cell morphology, a combined pattern of gene expression was detected in these p-iPS like cells (See Additional file 3). Those genes related with pluripotency (Oct4, Sox2, Nanog and Klf4) and genes related with differentiation to trophectoderm, primitive endoderm and mesoderm (Cdx2, Aromatase, Ple-1 and Esrrb; Sox17, Gata4 and Eomes; AFP respectively) were confirmed by RT-PCR of p-iPS like cells under KSR and FGF culture condition. Moreover, different colonies expressed different genes suggesting that they were not homogeneous under this culture condition (iPS like cells’ colony 1 and colony 2, See Additional file 3). AP positive cells were also detected at day 24 despite the loss of stem cell morphology (See Additional file 2, Fig. S2b). The above described results were similar for those cells cultured with and without sodium butyrate.

#### p-iPS like cells obtained in SB43 medium

**Fig. 1:**
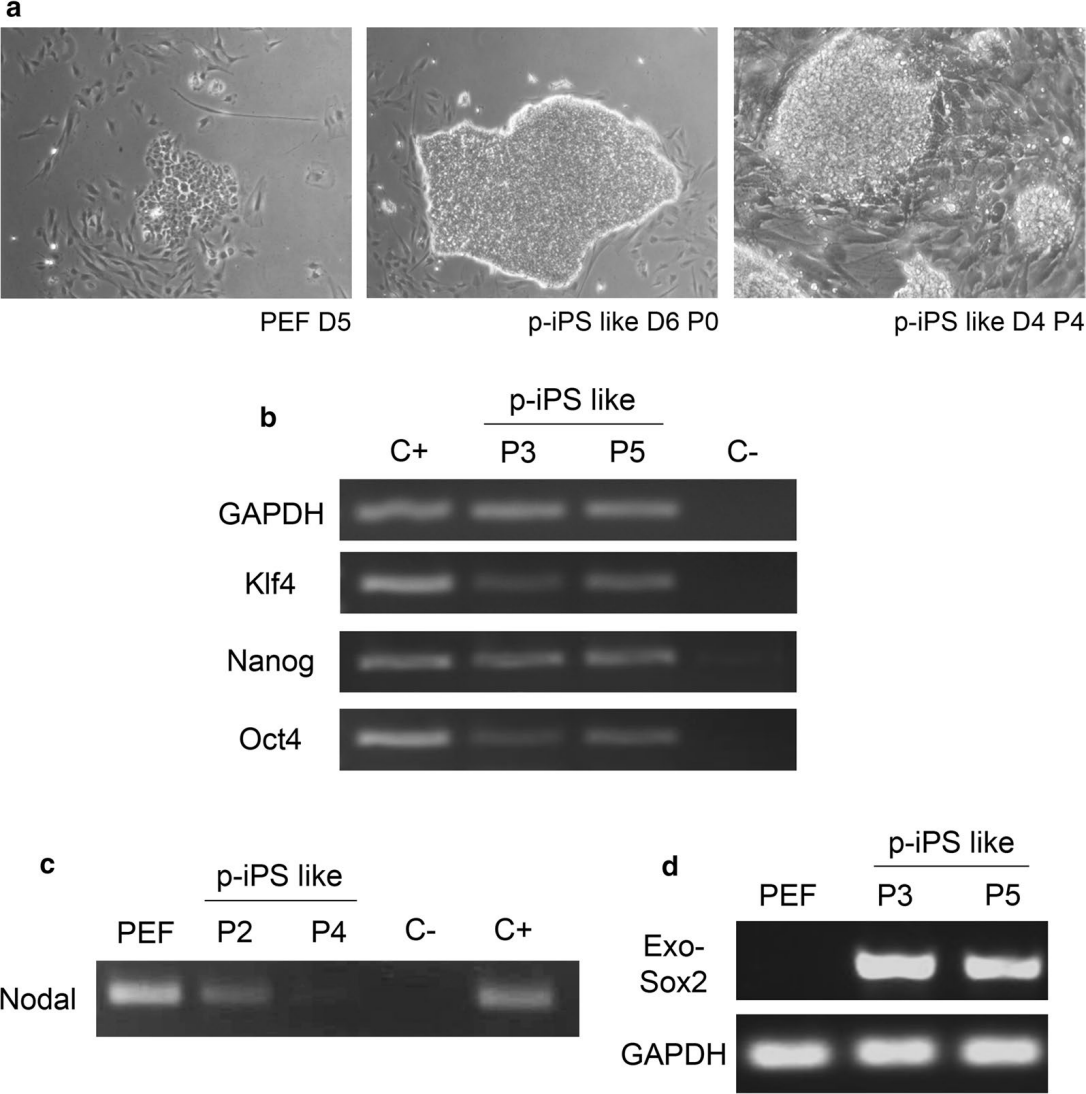
Porcine iPS like cells’ colonies from PEF transduced with STEMCCA cultured in SB43 medium. **a** Emerging colony at day 5 in Geltrex-coated dishes (left), at day 6 of culture (center), and flatten edged colonies of 4th passage after 4 days of culture (right); **b** RT-PCR analysis of gene expression of Klf4, Nanog and Oct4 from p-iPS-like cells’ colonies (C+ positive control, pool of porcine embryos; C− negative control, water; samples: p-iPS like cells from P3 and P5); **c** Nodal expression in PEF and progressive diminution of Nodal mRNA levels in iPS-like cells of passage 2–4. **d** Exo-Sox2 mRNA from STEMCCA was analysed by RT-PCR in p-iPSC like cells. Pool of porcine embryos (PEF) were used as a positive control, samples: p-IPSC P3 and p-iPSC P5

**Fig. 2:**
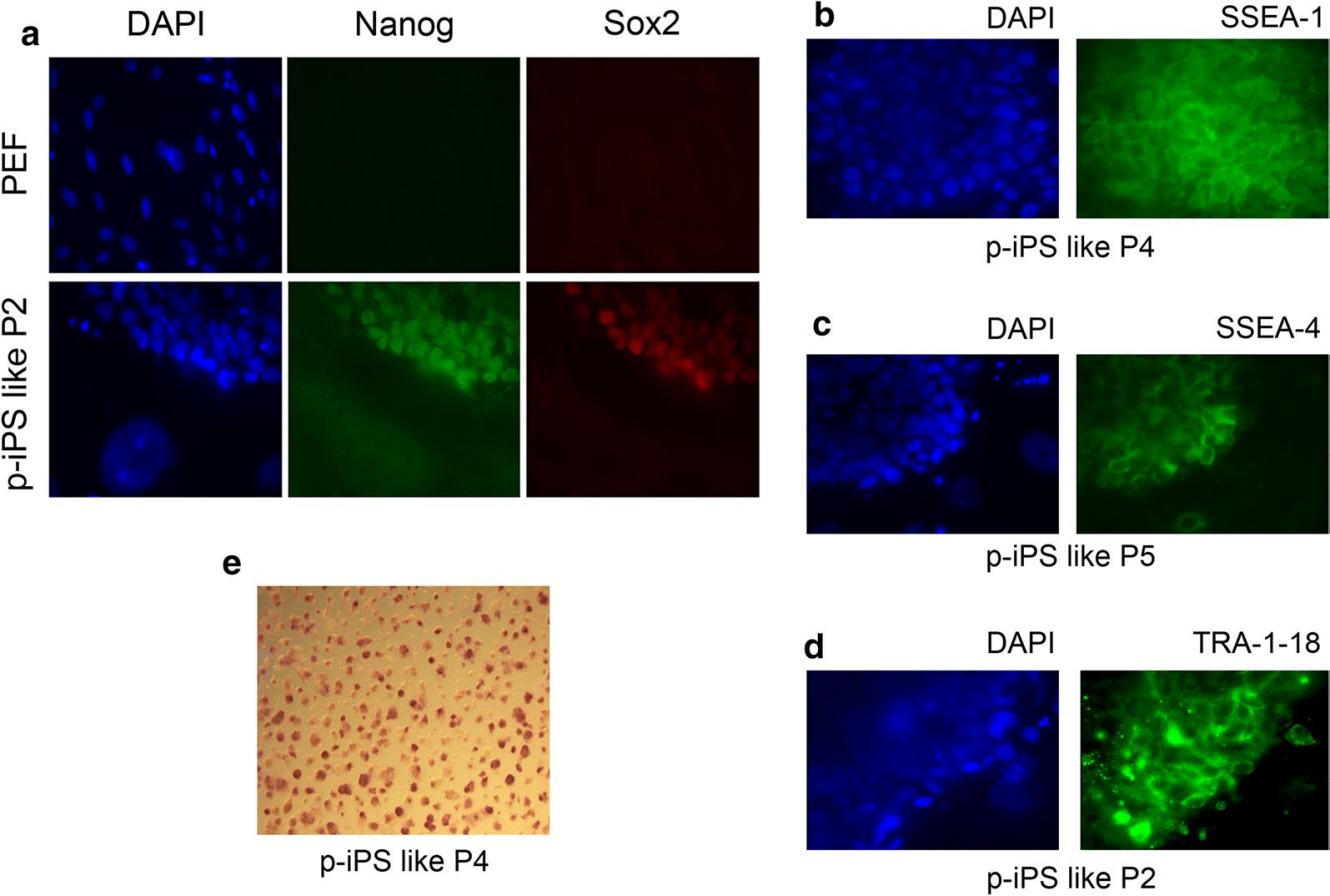
Pluripotency markers in p-iPS like cells. **a** Expression of pluripotency genes NANOG and SOX2 in p-iPS like cells P2; membrane markers of pluripotency SSEA1 (**b**), SSEA4 (**c**) and TRA-1-81 (**d**) from p-iPS like cells P4, P5 and P2 respectively. Nuclei were stained with DAPI (blue) and markers were detected by immunofluorescence followed by epifluorescence microscopy. **e** Alkaline phosphatase activity (AP) in p-iPS like cells of P4

In an attempt to avoid the loss of stem cells features through time in culture, we changed the culture conditions. We tested SB43 medium to reprogram and expand potentially reprogrammed cells. Colonies appeared from both species but at different periods: after 5 days from PEF and between days 15–18 in BEF. Cells were expanded manually and enzymatically and 8 clones of porcine iPS like cells were characterized and cryopreserved. The self- renewal feature was fully characterized in these cells by immunostaining of pluripotent stem cells markers, RT-PCR of endogenous genes and AP staining. Cells presented dome-shape morphology during early passages getting more flattened and losing the clear colony edge in later passages (Fig. 1a). We detected the expression of Oct4, Klf4, Nanog (Fig. 1b) and Nodal (Fig. 1c) by RT-PCR. We also detected the NANOG and SOX2 proteins by immunofluorescence (Fig. 2a), as well as the presence of membrane markers SSEA1, SSEA4, and TRA-1-81 (Fig. 2b–d) and AP activity (Fig. 2e). Finally, we detected the expression of h-OKSM from the STEMCCA vector not only in early but also in late passages of p-iPS like cells, confirming that cells were not fully reprogrammed. Silencing of exogenous genes is required for a proper differentiation [20, 21]. For this reason, pluripotency was not tested since we found that p-iPS like cells presented an active expression of exo-SOX2 from STEMCCA (Fig. 1d) and remained at least until passage 12.

#### b-iPS like cells obtained in SB43 medium

**Fig. 3:**
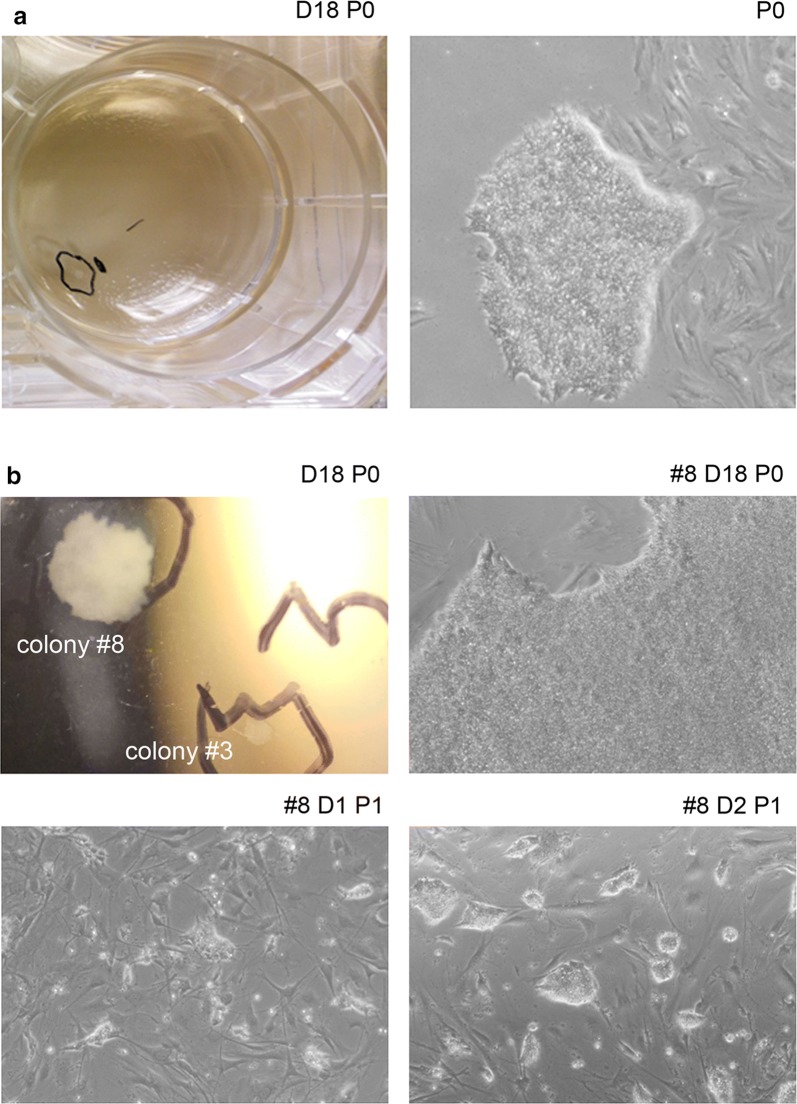
Bovine iPS like cells colonies. **a** b-iPS like cells’ colony #1 at Po, emerged in a 6-well plate coated with Geltrex and seeded at a density of 5000 cells/well of BEF. **b** b-iPS like cells’ colonies #3 and #8 from a plate seeded with 2500 cells/well of transduced BEF (top left); zoom of colony #8 (top right), expansion of colony #8, P1 at day 1 and day 2 (bottom)

Bovine iPS like cells colonies emerged at day 16 (Fig. 3), later than porcine. We amplified and cryopreserved eight colonies. In Fig. 3b, we showed colony #3 and #8. As we observed with p-iPS like cells, the use of SB43 medium generates homogeneous colonies also in bovine species. In a standard protocol of iPSC generation, a large colony as #8 would not be chosen for expansion. We demonstrated that with the use of the SB43 medium, selection of colonies is not a critical step for a successful expansion, as long as the colony is morphologically homogeneous (Fig. 3b). This colony could be expanded and cryopreserved, although full characterization of b-iPS like cells was not performed due to the expression of exogenous factors.

### Discussion

The technology to generate iPS cells in farm animals is under continuous revision and still requires further improvements, particularly to maintain pluripotency features in long term cultures without the expression of exogenous factors. Avoiding the use of integrative methods seemed a suitable option; however, we initially attempted to reprogram PEF and BEF cells with episomal vectors and obtained unsuccessful results (See Additional file 4). Then, we used the polycistronic STEMCCA cassette that encodes the four Yamanaka factors in the same lentiviral vector and integrates into the host genome. This approach ensures, at least, the expression of the four TFs in the same cell avoiding the disadvantage of random expression of the TFs and the lost of the vectors inherent to the episomal-based strategy [22]. Despite of detecting genomic integration of STEMCCA in BEF and PEF, our results showed that BEFs are more refractory to reprogramming in the basic culture conditions (DMEM/F12 plus KSR and FGF) than PEF. We tested two different sources of PEF (different foetuses) and five of BEF. We did not obtain colonies from BEF, whereas PEF colonies were abundant and appeared quickly after the second run of lentiviral transduction. p-iPS like colonies obtained were heterogeneous in KSR/FGF medium and started to differentiate during the first 18–20 days in culture. Moreover, they could not be manually expanded. At this time point, mouse iPSC reach the stabilization phase of the reprogramming process characterized by the independence of exogenous factors and can be expanded without losing the stem cell characteristic morphology [23]. With this approach, although we achieved a good delivery system, we couldn’t maintain the expression of endogenous pluripotency genes. Moreover, we did not obtain any colonies from BEF, suggesting a barrier in reaching the first step of the reprogramming process, which is the initiation phase, characterized by the mesenchymal to epithelial transition (MET) [23]. In human, small molecules were used in the defined medium SB43 to enhance MET transition and improve efficiency of h-iPS cells [18]. We reprogrammed BEF and PEF with an initial period post-transduction of 4–6 days in complete DMEM medium and then changing to SB43 medium. Under these conditions we obtained for the first time b-iPS like cells colonies, and as in human, we accelerated the kinetic of reprogramming in BEF, previously reported around day 30 [10, 11, 14, 16]. The kinetic of reprogramming PEF was also reduced from 14–21 days [8] to 5–7 days after the last transduction. In general, for both species, colonies were homogeneous in this culture condition, and could be expanded enzymatically by single cell disaggregation, a great advantage for these species that generally are expanded manually. Therefore, this procedure increased the efficiency of iPS like cells colony expansion. As p-iPS like cells were obtained before the ones from bovine, we fully characterized them by RT-PCR and IF of pluripotency markers. Moreover, we also detected the membrane markers SSEA1, SSEA4 and TRA-1-81 and the transcription factor NANOG, which was not included in the original reprogramming TFs, indicating the activation of the endogenous gene. We evaluated if exogenous TFs were silenced in early and late passages and, consistently with other recent reports [7, 24, 25] the iPS-like cells obtained in this work maintained expression of OSKM from the STEMCCA vector, indicating failure in silencing the exogenous TFs. The obstacles to obtain *bona fide* porcine iPS cells by using the protocol reported here are probably related to the inability of the cells to maintain the endogenous pluripotency TFs expression and to silence the transgenes in the stem cell culture medium. At the time of writing this manuscript, Ma et al. reported a paper PEF reprogramming with a Doxicyclin-inducible vector using the inhibitors CHIR, SB431542 and PD0325901, in combination with BMP4, SCF, IL-6 and human platelet lysates (PL). These culture conditions maintained the self-renewal and pluripotency of p-iPSCs [26]. However, so far, it has not been reported a suitable medium and the optimal culture conditions to sustain the self-renewal and pluripotency of b-iPSCs. Despite being more difficult to reprogram BEF than PEF, we agree that unsuccessful results for establishing b-iPSCs, independent of the source of cells used, has also been related with the inability of the cells of maintaining stemness features during long term culture in the culture context used [6].

Despite obtaining non-fully reprogrammed cells from BEF and PEF, we could elucidate some questions regarding reprogramming of farm animal cells with the experiments presented in this work. Remarkably, bovine fibroblasts are more difficult to reprogram than their counterpart in porcine species. All the experiments were done at the same time with both species (at least for triplicate each one) and it is a clear fact that BEFs present a higher struggle, probably due to their overgrowth during the time of reprogramming, which hampers the emergence of colonies [19]. Moreover, at the time of isolating and expanding BEF and PEF from foetuses, we detected differences in cell proliferation rates between both species. Therefore, both adjusting cell density and the number of transductions were critical for obtaining and detecting the first changes in morphology from BEF. Secondly, we could partially reprogram cells under feeder-free and serum-free conditions, using Geltrex as a matrix and KSR as serum replacement. Despite obtaining colonies in Geltrex-coated dishes we could not expand them in the same conditions, having to use MEFs as feeder layer from the second passage onwards. In addition, we used enzymes for clonal expansion of p-iPS and b-iPS single cell adding a ROCK inhibitor in the medium as was reported in a study with human iPSC [19]. This is important because it makes the protocol of expansion easier. Moreover, we could partially reprogram BEF using only the four Yamanaka TFs (OKSM) in its human versions and delivered by one lentiviral based vector. This had not been achieved before, mainly because it has been commonly reported the addition of other factors such as LIN28 and NANOG [4].

### Conclusion

Difficulties presented in b-iPS like and p-iPS like cells along the maturation phase suggest that modifications in the culture conditions may be necessary to transit to the stabilization phase of reprogramming. Taking into account that in the biology of the pluripotent cells this is a critical step, we speculate that in the last phase of the reprogramming process the endogenous pluripotency genes are responsible for maintaining stemness. More studies are required to unravel molecular mechanisms involved in the establishment of the pluripotent cell population during embryogenesis. To our knowledge, maintenance of pluripotency in vitro is still a bottle neck in farm animals. Future studies need to concentrate efforts in deepen the understanding of the biology of pluripotency in pig and bovine embryos so that we can define media formulations and reprogramming cocktails that may be suitable for the generation and stable propagation of iPSCs from livestock.

### Limitations

Cells obtained were not fully characterized. We did not perform differentiation analysis since cells were partially reprogrammed.

## Additional files

**Additional file 1.** doc, Supplementary Methods. Additional information about the experimental procedures and primers sequences (Supplementary Table 1 and Supplementary Table 2 and their legends). This file also contains Supplementary Fig. 1 and its legend.

**Additional file 2.** doc, Supplementary Fig. 2, this file contains the figure and its legend.

**Additional file 3.** doc, Supplementary Fig. 3, this file contains the figure and its legend.

**Additional file 4.** doc, Supplementary Results, this file contains Supplementary Results, Supplementary Fig. 4 and its legend and Supplementary Table 3 and its legend.

## Abbreviations

2i: MEK and GSK3β inhibitors
AP: alkaline phosphatase
BEF: bovine foetal fibroblast
FCS: foetal calf serum
FGF: fibroblast growth factor
iPSC: induced pluripotent stem cells
IWR1: Wnt inhibitor
KSR: knock out serum replacement
MEF: mouse embryonic fibroblast
MOI: multiplicity of infection
NaB: sodium butyrate
OKSM: Oct4, Klf4, Sox2 and c-Myc
PEF: porcine foetal fibroblast
RPM: reprogram medium
TFs: transcription factors

## References

[1] Takahashi K, Yamanaka S (2006). Induction of pluripotent stem cells from mouse embryonic and adult fibroblast cultures by defined factors. Cell.

[2] Takahashi K, Tanabe K, Ohnuki M, Narita M, Ichisaka T, Tomoda K, Yamanaka S, Jaenisch R, Thomson JA, Jaenisch R (2007). Induction of pluripotent stem cells from adult human fibroblasts by defined factors. Cell.

[3] Yu J, Vodyanik MA, Smuga-Otto K, Antosiewicz-Bourget J, Frane JL, Tian S, Nie J, Jonsdottir GA, Ruotti V, Stewart R, Slukvin II, Thomson JA (2007). Induced pluripotent stem cell lines derived from human somatic cells. Science.

[4] Ogorevc J, Orehek S, Dovč P (2016). Cellular reprogramming in farm animals: an overview of iPSC generation in the mammalian farm animal species. J Anim Sci Biotechnol..

[5] Nowak-Imialek M, Niemann H (2012). Pluripotent cells in farm animals: state of the art and future perspectives. Reprod Fertil Dev..

[6] Ezashi T, Yuan Y, Roberts RM (2016). Pluripotent stem cells from domesticated mammals. Annu Rev Anim Biosci..

[7] Zhao L, Wang Z, Zhang J, Yang J, Gao X, Wu B, Zhao G, Bao S, Hu S, Liu P, Li X (2017). Characterization of the single-cell derived bovine induced pluripotent stem cells. Tissue Cell.

[8] Hanning W, Yangli P, Ning L, Jianyong H (2014). Progress, problems and prospects of porcine pluripotent stem cells. Front Agric Sci Eng..

[9] Congras A, Barasc H, Canale-Tabet K, Plisson-Petit F, Delcros C, Feraud O, Oudrhiri N, Hadadi E, Griscelli F, Bennaceur-Griscelli A, Turhan A, Afanassieff M, Ferchaud S, Pinton A, Yerle-Bouissou M, Acloque H (2016). Non integrative strategy decreases chromosome instability and improves endogenous pluripotency genes reactivation in porcine induced pluripotent-like stem cells. Sci Rep..

[10] Cao H, Yang P, Pu Y, Sun X, Yin H, Zhang Y, Zhang Y, Li Y, Liu Y, Fang F, Zhang Z, Tao Y, Zhang X (2012). Characterization of bovine induced pluripotent stem cells by lentiviral transduction of reprogramming factor fusion proteins. Int J Biol Sci..

[11] Sumer H, Liu J, Malaver-Ortega LF, Lim ML, Khodadadi K, Verma PJ (2011). NANOG is a key factor for induction of pluripotency in bovine adult fibroblasts. J Anim Sci..

[12] Lei LEI, Li LEI, Du F, Chen C, Wang H (2013). Monitoring bovine fetal fibroblast reprogramming utilizing a bovine NANOG promoter-driven EGFP reporter. System.

[13] Heo YT, Quan X, Xu YN, Baek S, Choi H, Kim NH, Kim J (2015). CRISPR/Cas9 nuclease mediated gene knockin in bovine induced pluripotent cell. Stem Cells Dev..

[14] Han X, Han J, Ding F, Cao S, Lim SS, Dai Y, Zhang R, Zhang Y, Lim B, Li N (2011). Generation of induced pluripotent stem cells from bovine embryonic fibroblast cells. Cell Res..

[15] Huang B, Li T, Alonso-Gonzalez L, Gorre R, Keatley S, Green A, Turner P, Kallingappa PK, Verma V, Oback B (2011). A virus-free poly-promoter vector induces pluripotency in quiescent bovine cells under chemically defined conditions of dual kinase inhibition. PLoS ONE..

[16] Talluri TR, Kumar D, Glage S, Garrels W, Ivics Z, Debowski K, Behr R, Niemann H, Kues WA (2015). Derivation and characterization of bovine induced pluripotent stem cells by transposon-mediated reprogramming. Cell Reprogram..

[17] Polo JM (2014). Phases of reprogramming. Stem Cell Res..

[18] Lin T, Ambasudhan R, Yuan X, Li W, Hilcove S, Abujarour R, Lin X, Hahm HS, Hao E, Hayek A, Ding S (2009). A chemical platform for improved induction of human iPSCs. Nat Methods..

[19] Beers J, Gulbranson DR, George N, Siniscalchi LI, Jones J, Thomson JA, Chen G (2012). Passaging and colony expansion of human pluripotent stem cells by enzyme-free dissociation in chemically defined culture conditions. Nat Protoc.

[20] Hall VJ, Kristensen M, Rasmussen MA, Ujhelly O, Dinnyés A, Hyttel P (2012). Temporal repression of endogenous pluripotency genes during reprogramming of porcine induced pluripotent stem cells. Cell Reprogram..

[21] Zhou Y, Zeng F (2013). Integration-free methods for generating induced pluripotent stem cells. Genom Proteom Bioinform..

[22] Okita K, Matsumura Y, Sato Y, Okada A, Morizane A, Okamoto S, Hong H, Nakagawa M, Tanabe K, Tezuka K, Shibata T, Kunisada T, Takahashi M, Takahashi J, Saji H, Yamanaka S (2011). A more efficient method to generate integration-free human iPS cells. Nat Methods.

[23] González F, Huangfu D (2016). Mechanisms underlying the formation of induced pluripotent stem cells. Wiley Interdiscip Rev..

[24] Wu Y, Li O, He C, Li Y, Li M, Liu X, Wang Y, He Y (2017). Generation and characterization of induced pluripotent stem cells from guinea pig fetal fibroblasts. Mol Med Rep..

[25] Zhang W, Wang H, Zhang S, Zhong L, Wang Y, Pei Y, Han J, Cao S (2018). Lipid supplement in the cultural condition facilitates the porcine iPSC derivation through cAMP/PKA/CREB signal pathway. Int J Mol Sci..

[26] Ma Y, Yu T, Cai Y, Wang H (2018). Preserving self-renewal of porcine pluripotent stem cells in serum-free 3i culture condition and independent of LIF and b-FGF cytokines. Cell Death Discov..

