# Supplementary material for: Exogenous human OKSM factors maintain pluripotency gene expression of bovine and porcine iPS-like cells obtained with STEMCCA delivery system
Source: BMC Res Notes. 2018 Jul 27;11:509. doi: 10.1186/s13104-018-3627-8 (PMC6062933; doi:10.1186/s13104-018-3627-8)
Supplement: Supplementary file 1 — Additional file 1. doc, Supplementary Methods. Additional information about the experimental procedures and primers sequences (Supplementary Table 1 and Supplementary Table 2 and their legends). This file also contains Supplementary Fig. 1 and its legend. [file 13104_2018_3627_MOESM1_ESM.docx]

**Supplementary Methods**

**Cell culture**

Primary fibroblast from bovine and porcine foetuses (BEF and PEF respectively) were previously obtained at the laboratory as described in [1]. Cells were cultured in complete DMEM with 10% of foetal calf serum (FCS, GIBCO) and supplements.

**Preparation of lentiviral particles**

Lentiviral particles were produced using the pHAGE-EF1a-STEMCCA vector and helper plasmids as previously described [2], [3], with minor modifications. Briefly, the two-plasmid transfection system from ADDGENE (pPAX2 and PMD2G) was introduced in 293T cells using FUGENE 6 transfection reagent (ROCHE) according to the manufacturer’s recommendations. Optimal transfection efficiency was obtained using a FUGENE 6 Reagent: DNA ratio of 3:1. Virus-containing supernatants were collected 24, 36, 72 and 96 h after transfection and filtered through a 0.45 μm pore size syringe filter. Lentiviral vector STEMCCA was harvested by ultracentrifugation and then aliquoted and cryopreserved at -80°C until using it.

**Transduction and reprogramming of PEF and BEF in KSR + FGF medium**

BEF and PEF cells were transduced twice starting a day after being plated at two densities: 40.000 and 80.000 cells per a six well for BEF; and 50.000 and 100.000 cells per a six well for PEF (taking into account the differences in the cells proliferation rate between species, detected previously). It is recommended in Mostosvlasky’s protocol to plate cells in a density that allow them to reach 90-95% of confluence between day 5-6 [4]. We tested three different MOI of STEMCCA concentrated supernatant (low: 0.3 µl of STEMCCA, medium: 1.5 µl and high 7.5 µl). At day 5 in culture, cells reached 90-95 % of confluence and 40.000 transduced BEF and 100.000 transduced PEF were plated onto a 10 cm dish with mitomycin treated MEFs (2 x 10^6^ m-MEFs per p100 plate). The seeding density of transduced BEF and PEF was previously established in our laboratory. We considered growth rate of each cell type to avoid premature over confluence while waiting the reprogramming of the cells. For each MOI we plated two culture conditions: DMEM/F12 (GIBCO) with 20 % of KSR and 20 ng/ml of FGF with and without 0.1 mM sodium butyrate (NaB). Medium was changed every 2 days.

**Transduction and reprogramming of PEF and BEF in SB43 medium**

**
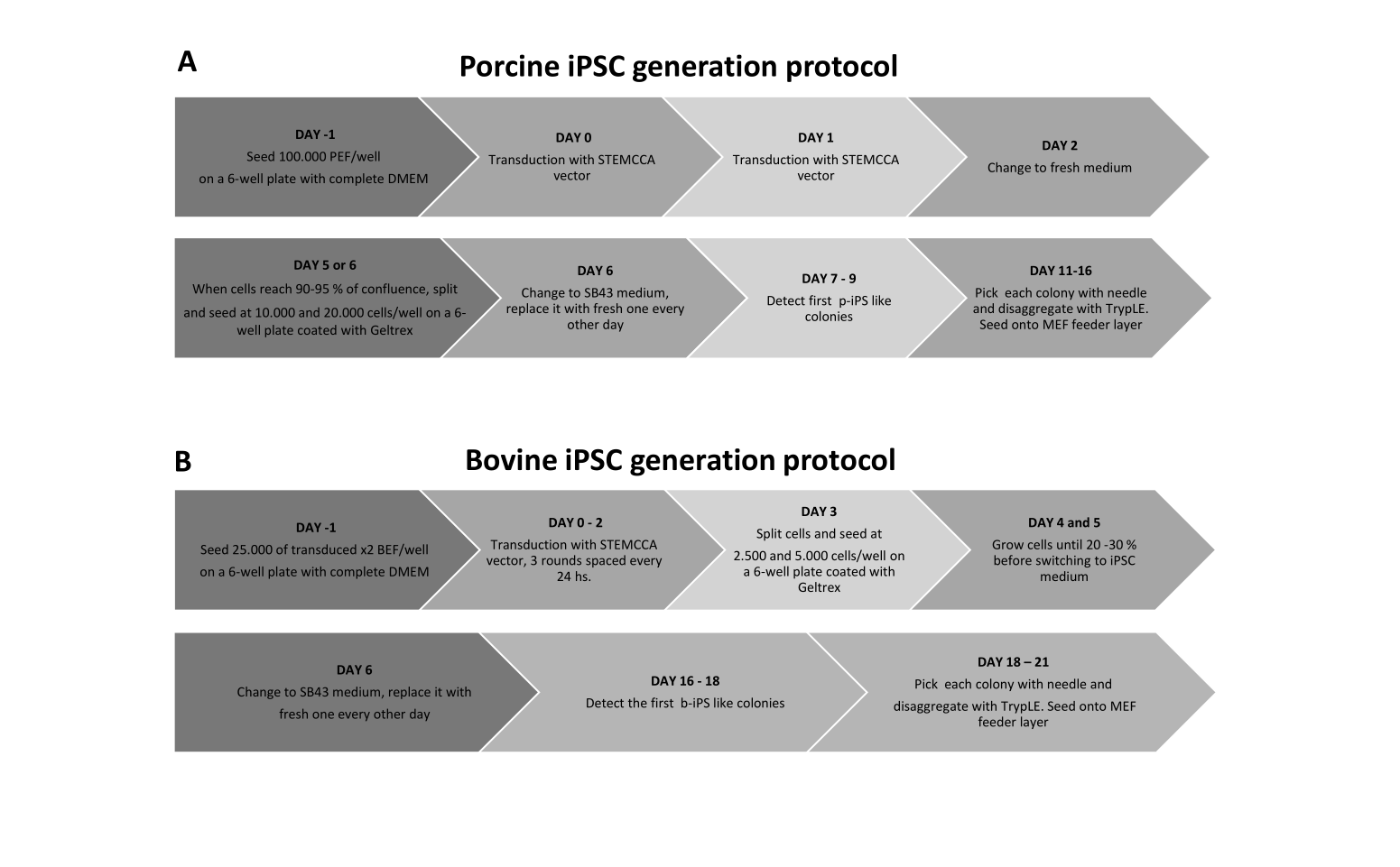
**

**Supplementary Figure 1: iPS-like cells derivation from porcine and bovine fibroblasts in chemically defined medium SB43.** (A) A brief summary of the steps described in the protocol to generate porcine iPS-like cells. (B) Schematic representation of the modified protocol for bovine iPS-like cells generation.

**Characterization of iPS-like cells**

**Immunofluorescence and Alkaline phosphatase**

BEF, PEF and iPSC-like were cultured in each specific condition previously described for at least three passages. Then, they were fixed, permeabilized and incubated for 1 hour with the following primary antibodies: NANOG (1∶100; rabbit polyclonal, PEPROTECH, 500-P236), SOX2 (1∶200; goat polyclonal, SANTA CRUZ BIOTECHNOLOGY, SC17320), TRA-1-81 (1∶10; mouse polyclonal, Hybridoma Bank, Iowa City), SSEA-1 (1∶10; mouse polyclonal, Hybridoma bank, Iowa City) and SSEA-4 (1∶10; mouse polyclonal, HYBRIDOMA BANCK, IOWA CITY), and detected with appropriate secondary antibody as previously described [1]. Nuclei were stained with DAPI according to the manufacturer’s instructions (FLUOROSHIELD, SIGMA). Images were acquired with a fluorescence microscope.

Alkaline phosphatase (AP) activity was determined with the AP kit (SIGMA ALDRICH) following the manufacturer’s instructions.

**RNA Isolation, retro- transcription and Polymerase Chain Reaction**

RNA isolation was carried out using RNeasy KIT (QIAGEN) following the manufacturer’s instructions. RNA reverse transcription was performed using OMNISCRIPTSYNTHESIS KIT (QIAGEN). End-point PCR was performed with READYMIX (SIGMA ALDRICH) and 0.4 µM of each primer. PCR products were run in 1.5 % agarose gel to determine the amplicon size. Quantitative RT-PCR (qRT-PCR) was performed using SYBR green mix (ROCHE) and 0.25 nM of each primer. For each gene, the analysis was performed in triplicate.

RT-PCR protocol included an initial step of 95 °C (5 min), followed by 45 cycles of 10 sec at 95 °C, 15 sec at 60 °C and a primer extension step of 20 sec at 72 °C. Fluorescence data were acquired at 72 °C. Melting-curve analysis to confirm product specificity was performed immediately after amplification and the amplicon size was verified by electrophoresis. The relative expression of the target gene was normalized with GAPDH and a calibrator sample. Sequence accession numbers were obtained from NCBI, Ensemble and TGI databases. Primers used in this study are listed in Supplementary Table 1 .

**Supplementary Table 1**

List of primers used in this study (for porcine cells)

| Gene | Primer sequence Fw  Primer sequence Rv | Amplicon  (bp) |
| --- | --- | --- |
| Gapdh | GGG CAT GAA CCA TGA GAA GT  GTC TTC TGG GTG GCA GTG AT | 162 |
| Oct3/4 | GCA AAC GAT CAA GCA GTG A  GGT GAC AGA CAC CGA GGG AA | 201 |
| Nanog | AAG TAC CTC AGC CTC CAG CA  GGC ATC CTT GGT GAT AGG AA | 202 |
| Sox2 | AAGAGAACCCCAAGATGCACAACT  GCT TGG CCT CGT CGA TGA AC | 105 |
| Klf4 | CCA TGG GCC AAA CTA CCC AC  TGG GGT CAA CAC CAT TCC GT | 154 |
| Cdx2 | AGA ACC CCC AGG TCT CTG TCT T  CAG TCC GAA ACA CTC CCT CAC A | 101 |
| Eomes | TAC CAA CCA CGT CTG CAC AT  GGA AGC GGT GTA CAT GGA GT | 210 |
| Gata4 | AAT CGA AGA CGT CAG CAG GT  GGC CAG ACA TGG CAC TAA CT | 195 |
| Sox17 | CTT CAT GGT GTG GGC TAA GG  TTG TAG TTG GGG TGG TCC TG | 186 |
| Nodal | CGT CTC CAG ATG GAC CTG TT  CTG CTC TGG AGA GAG GTT GG | 222 |
| Ple-1 | CTG GCA AAA AGC AGA CAT GA  AGC TGT AGC CTC CTT GTG GA | 163 |
| AFP | AGA TGC CCA TAA ACC CTG GT  CCA GTA GAC CAG AGA AAT CTG CA | 270 |
| Esrrb | AGC CCG TAC CTG AGC TTA CA  CAA CCA ATG ATG ACC ACG AG | 191 |
| Exo Sox2/IRES | TGG CTC TCC TCA AGC GTA TT  GCT TAG CCT CGT CGA TGA AC | 482 |

##### **Detection of genomic insertion of STEMCCA**

Genomic DNA was extracted from iPS like cells using a kit (QIAGEN) and following the manufacturer’s instructions. End-point PCR was performed with READYMIX (SIGMA ALDRICH) as described before with primers described in Supplementary Table 2. PCR products were run in 1 % agarose gel to determine the amplicon size.

End-PCR protocol included an initial step of 94 °C (3 min), followed by 35 cycles of 45 sec at 94 °C, 30 sec at 61 °C and a primer extension step of 1 min and 30 sec at 72 °C. Final step was 10 min at 72 °C.

**Supplementary Table 2**

Sequence of primers used for genomic detection of STEMCCA by PCR (bp: base pair, Fw: Forward, Rv: reverse)

| **Primers** | **Amplicon**  **(bp)** | **Primer sequence Fw**  **Primer sequence Rv** |
| --- | --- | --- |
| **OCT4_KLF4**  **Bridge** | 560 | CAACGAGAGGATTTTGAGGCTGC  ATCGTTGAACTCCTCGGTCTCTCT |

**References**

[1] A. Rodríguez, C. Allegrucci, and R. Alberio, “Modulation of pluripotency in the porcine embryo and iPS cells.,” *PLoS One*, vol. 7, no. 11, p. e49079, 2012.

[2] C. A. Sommer, M. Stadtfeld, G. J. Murphy, K. Hochedlinger, D. N. Kotton, and G. Mostoslavsky, “Induced Pluripotent Stem Cell Generation Using a Single Lentiviral Stem Cell Cassette,” *Stem Cells*, vol. 27, no. 3, pp. 543–549, Mar. 2009.

[3] D. N. Kotton, G. Mostoslavsky, and G. J. Murphy, “Packaging of VSV-G Pseudotyped Lentivirus by 5 Plasmid Co- Transfection of 293T cells.” [Online]. Available: http://www.bu.edu/dbin/stemcells/files/Protocol 1- Lentivirus Packaging by 293T Transfection.pdf. [Accessed: 28-Jun-2018].

[4] D. N. Kotton, G. Mostoslavsky, and G. J. Murphy, “Human Fibroblast Reprogramming to make human iPS Cells (iPSC).” [Online]. Available: http://www.bu.edu/dbin/stemcells/files/Human iPS cell generation with hSTEMCCA.pdf. [Accessed: 28-Jun-2018].
