# Supplementary material for: Exogenous human OKSM factors maintain pluripotency gene expression of bovine and porcine iPS-like cells obtained with STEMCCA delivery system
Source: BMC Res Notes. 2018 Jul 27;11:509. doi: 10.1186/s13104-018-3627-8 (PMC6062933; doi:10.1186/s13104-018-3627-8)
Supplement: Supplementary file 2 — Additional file 2. doc, Supplementary Fig. 2, this file contains the figure and its legend. [file 13104_2018_3627_MOESM2_ESM.docx]

**
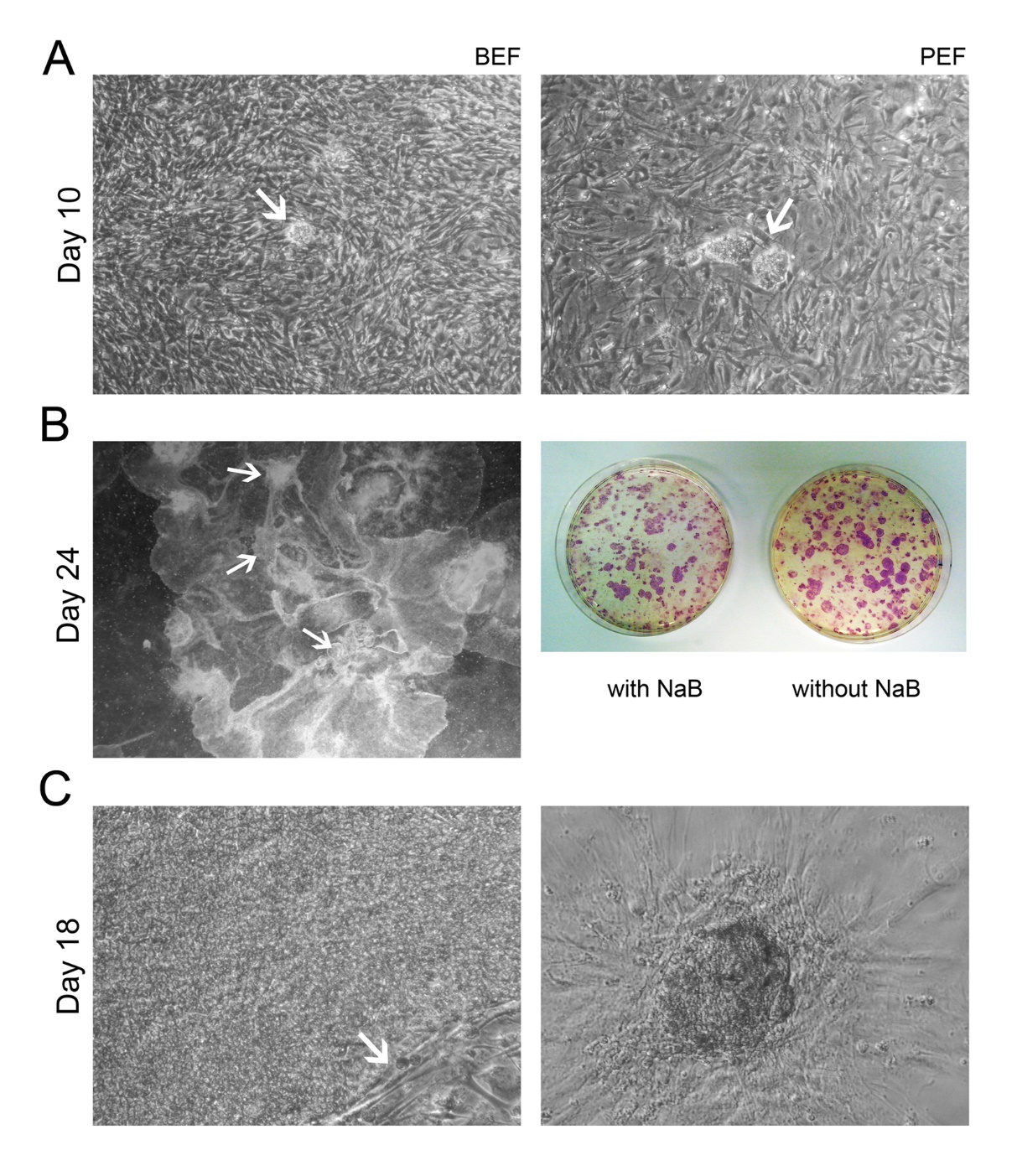
**

**Supplementary Figure 2: iPS-like cells from BEF and PEF transduced with STEMCCA vector cultured in DMEM/F12 with KSR and FGF.** (A) Emerging colonies (arrows) at day 10 post- transduction from BEF (left) and PEF (right). (B) p-iPS like cells at day 24 post-transduction. Arrows indicate globular structures similar to extraembryonic lineage tissue (left) and AP activity with or without 0.1 mM sodium butyrate, NaB (right). (C) p-iPS like cells’ colony before (left) and after (right) expansion at day 18. Arrow indicates the edge between the selected colony and the MEF feeder layer.
