# Supplementary material for: Exogenous human OKSM factors maintain pluripotency gene expression of bovine and porcine iPS-like cells obtained with STEMCCA delivery system
Source: BMC Res Notes. 2018 Jul 27;11:509. doi: 10.1186/s13104-018-3627-8 (PMC6062933; doi:10.1186/s13104-018-3627-8)
Supplement: Supplementary file 3 — Additional file 3. doc, Supplementary Fig. 3, this file contains the figure and its legend. [file 13104_2018_3627_MOESM3_ESM.docx]

**
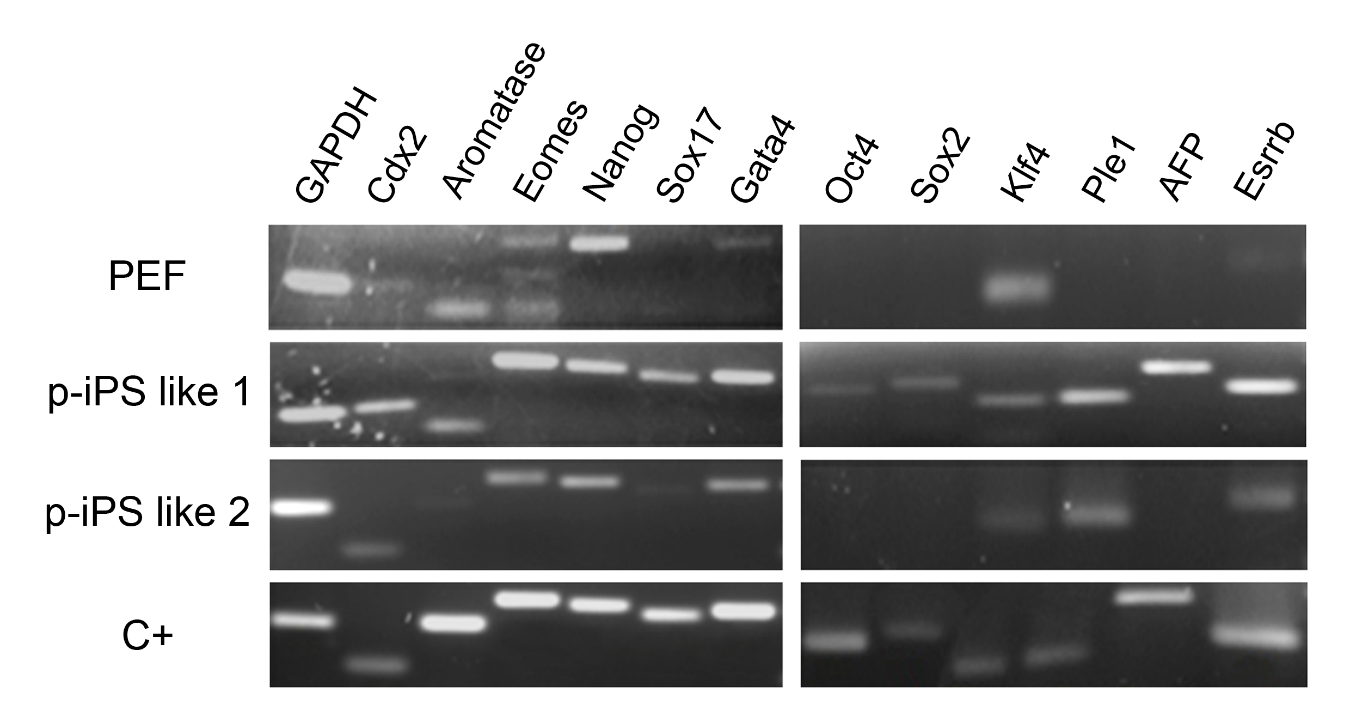
**

**Supplementary Figure 3: Expression of pluripotency and differentiation-related genes in PEF and p-iPS-like cells at day 24.** mRNA levels of the indicated genes were analysed by RT-PCR and visualized by agarose gel electrophoresis. Positive controls (C+) were porcine trophectoderm c-DNA for Cdx2, Aromatase, Ple-1 and Esrrb; porcine primitive endoderm c-DNA for Sox17, Gata4 and Eomes; porcine epiblast c-DNA for Oct4, Sox2, Nanog y Klf4; porcine liver for AFP.
