# Supplementary material for: Exogenous human OKSM factors maintain pluripotency gene expression of bovine and porcine iPS-like cells obtained with STEMCCA delivery system
Source: BMC Res Notes. 2018 Jul 27;11:509. doi: 10.1186/s13104-018-3627-8 (PMC6062933; doi:10.1186/s13104-018-3627-8)
Supplement: Supplementary file 4 — Additional file 4. doc, Supplementary Results, this file contains Supplementary Results, Supplementary Fig. 4 and its legend and Supplementary Table 3 and its legend. [file 13104_2018_3627_MOESM4_ESM.docx]

**Supplementary Results**

Our experiments with foetal fibroblasts from bovine and porcine skin tissues required setting up the transfection protocol. Nucleofection with Amaxa and transfection with Fugene were tested at the same time for introducing the vectors into the host cells (data not shown). Efficiency of transfection was determined using a GFP episomal vector and quantification of the percentage of GFP-expressing cells was performed by FACS (Supplementary Figure 4a). The results obtained demonstrated that nucleofection protocols are optimized to use them in other species, but in farm animals we obtained lower efficiency than Fugene (11 % ± 5 and 26 % ± 7, respectively). Fugene based transfection protocols allowed us to optimize some parameters such as initial density of host cells, DNA:Fugene ratio, and amount of total DNA transfected.


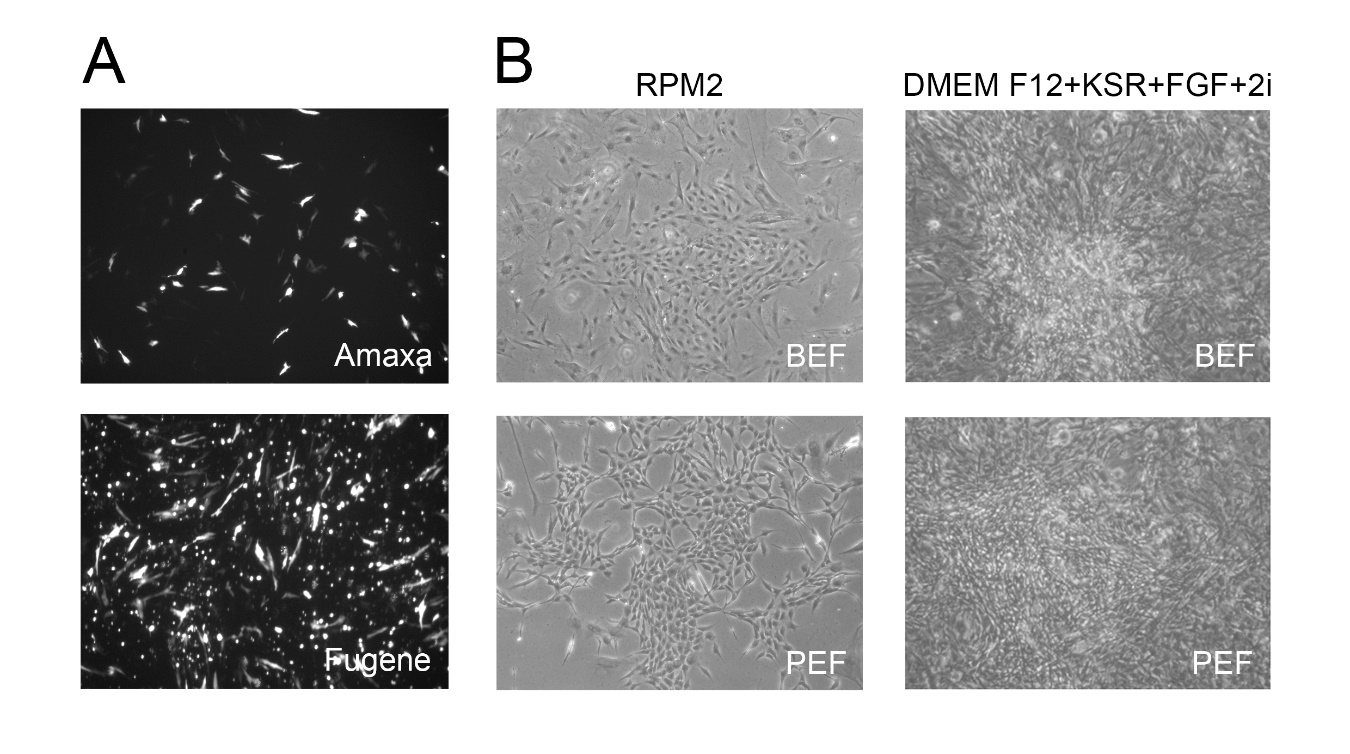


**Supplementary Figure 4: Reprogramming protocol with episomal vectors in bovine and porcine fibroblasts.** (A) BEF were transfected with a GFP expression vector by nucleofection with Amaxa or Fugene, as indicated. Images were taken at 48 h post- transfection with a fluorescence microscope. (B) BEF and PEF on day 30 after the episomal transfection with Fugene, in a well coated with Geltrex and cultured with RPM2 (left panels) or with DMEM/F12, 20 % KSR, FGF (20 ng/ml), 1 µM PD32 and 3 µM de CHIR (right panels).

After setting up the transfection with Fugene, we performed many attempts to reprogram bovine and porcine fibroblasts with a system that avoids vector chromosome integration (Supplementary Table 3) and detected no changes in cell morphology under any condition. Additionally, cells stopped growing and did not complete reprogramming in culture media without KSR (Supplementary Figure 4b, left panels). In media supplemented with KSR (DMEM/F12, 20% KSR, FGF and 2i) cells grew extensively (Supplementary Figure 4b, right panels). In our multiple attempts cells could not be successfully reprogrammed by episomal vector transfection even though adjusting cell density and serial (one, two and four) transfections. We speculated that the expression levels of OKSM factors were not sufficient to complete reprogramming, therefore we tested the same culture media but with cells transduced with the lentiviral vector h-STEMCCA [1].

**Supplementary Table 3:**

Episomal vector transfection failed to achieve a proper reprogramming of bovine and porcine fibroblasts

| **Donor Cells** | **Density of cells (cells/plate)** | **Method of transfection** | **Substrate** | **Medium** | **Results** |
| --- | --- | --- | --- | --- | --- |
| Bovine (BEF) or Porcine (PEF)  Fibroblast | 300.000,  Previously to transfection | 4ug  1µg/vector  8 µl of fugene  1x, 2x | Geltrex  2 plates of six wells | RPM1  (until 20% of confluence)  RPM2  (until colonies)  E8 (expansion) | No changes.  Cells arrested |
|  | 100.000, transfected  in a p100 | 4ug  1µg/vector  8 µl of Fugene  1x, 2x, 4x | MEF  2x10^6^/p100 | DMEM/F12 20% KSR + 2i +  20 ng/ml FGF  +/- NaB | No changes.  Fibroblast overgrowth |
|  | 100.000, transfected in a p100 | 4ug  1µg/vector  8 µl of Fugene  4x | MEF  2x10^6^/p100 | SB43  or  N2B27 with FGF + IWR1 | No changes after 30 days |

**References**

[1] C. A. Sommer, M. Stadtfeld, G. J. Murphy, K. Hochedlinger, D. N. Kotton, and G. Mostoslavsky, “Induced Pluripotent Stem Cell Generation Using a Single Lentiviral Stem Cell Cassette,” *Stem Cells*, vol. 27, no. 3, pp. 543–549, Mar. 2009.
